# The Influence of Gold Nanoparticles Addition on Sugarcane Leaves-Derived Silica Xerogel Catalyst for the Production of Biodiesel

**DOI:** 10.3390/gels11030153

**Published:** 2025-02-20

**Authors:** Ncamisile Nondumiso Maseko, Dirk Enke, Pius Adewale Owolawi, Samuel Ayodele Iwarere, Oluwatobi Samuel Oluwafemi, Jonathan Pocock

**Affiliations:** 1 Discipline of Chemical Engineering, University of KwaZulu-Natal, 238 Mazisi Kunene Road, Glenwood, Durban 4041, South Africa; dirk.enke@uni-leipzig.de (D.E.); pocockj@ukzn.ac.za (J.P.); 2 Institute of Chemical Technology, Universität Leipzig, Linnéstr. 3, 04103 Leipzig, Germany; 3 Department of Computer Systems Engineering, Tshwane University of Technology, Pretoria 0152, South Africa; owolawipa@tut.ac.za; 4 Department of Chemical Engineering, University of Pretoria, Lynnwood Road, Hatfield, Pretoria 0028, South Africa; samuel.iwarere@up.ac.za; 5 Department of Chemical Sciences (Formerly Applied Chemistry), University of Johannesburg, Doornfontein Campus, Doornfontein 2028, South Africa; oluwafemi.oluwatobi@gmail.com; 6 Centre for Nanomaterials Science Research, University of Johannesburg, Johannesburg 2000, South Africa

**Keywords:** gold nanoparticles, silica xerogel, catalyst support, biodiesel, transesterification, sugarcane leaves

## Abstract

Biodiesel was produced via transesterification of canola oil in the presence of a silica xerogel catalyst with deposited gold nanoparticles. The silica-gold catalyst was produced in situ, where gold metal was added to a sodium silicate solution; subsequently, gold nanoparticles were synthesised within the solution. The sodium silicate-gold nanoparticles solution was then turned into a silica-gold gel at pH 8.7 and later dried to form silica-gold nanoparticles xerogel. The produced silica-gold nanoparticles xerogel was characterised by X-ray diffraction (XRD), X-ray fluorescence (XRF), transition electron microscopy (TEM), and nitrogen physisorption. The gel had a silica content of 91.6 wt% and a sodium content of 6.4 wt%, with the added gold content being 99.5% retained. The biodiesel produced in the presence of silica-gold nanoparticles xerogel was characterised by gas chromatography-mass spectroscopy (GC-MS) and its physical properties, such as density, kinematic viscosity, flash point, pour point, and cloud point, were also determined. The silica-gold nanoparticles xerogel catalyst remained solid throughout its usage without leaching into the reaction medium. The produced biodiesel contained mostly monounsaturated fatty acid methyl esters and had a yield of 99.2% at optimum reaction conditions.

## 1. Introduction

**Figure 1:**
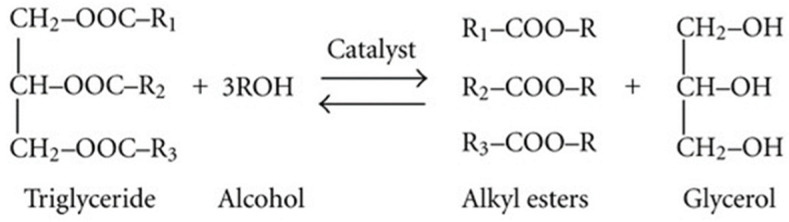
Transesterification schematic reaction.

Today’s world is facing an energy crisis as the finite resources of fossil fuels are unlikely to meet the continuously growing demand for energy. The exhaustion of fossil fuel reserves and the escalating costs of oil and its byproducts have prompted significant apprehension due to the heightened environmental pollution associated with fossil fuel combustion. As a result, humanity is increasingly motivated to explore alternative energy sources that offer greater sustainability [1]. Biodiesel is one of these alternative energies and its presence has noticeable effects on the world of biofuels [2]. The classification of biodiesel as one of the most preferred types of biofuel is primarily attributed to its low sulphur content, reduced toxicity, elevated cetane number, properties similar to fossil diesel, high combustion efficiency, elevated flash point, and most significantly, its lower carbon emissions. Biodiesel is generally manufactured by the transesterification of vegetable oils using methanol as a solvent. This procedure occurs with the aid of either a base or an acid catalyst [3], as indicated in Figure 1.

In the conventional transesterification process, homogeneous base catalysts, including sodium hydroxide, potassium hydroxide, or alkoxides, are typically favoured due to their considerably higher activity and reaction rates under moderate conditions compared with acid-base catalysts [4,5]. Homogeneous catalysts, however, present significant disadvantages, including their lack of environmental friendliness, the generation of wastewater during downstream processing [6], the necessity for costly equipment due to their corrosive properties [4], and lastly, the complexity involved in separating them from the reaction media [5]. Consequently, research efforts have increasingly focused on the application of heterogeneous catalysts in biodiesel production. These catalysts are in a different phase from raw materials and products. Heterogeneous catalysts are easy to separate from a reaction medium, non-corrosive, and are effective [7]. Therefore, heterogeneous catalysts are cost-effective and environmentally friendly [8]. Alkaline earth metal oxides, including calcium oxide (CaO), magnesium oxide (MgO), and strontium oxide (SrO), which exhibit significant basic strength, have been utilised in the biodiesel production process [9] at reduced reaction temperatures and pressures. Calcium oxide produced from different biological wastes has been one of the most researched solid catalysts in the literature. Chong et al. [10] derived CaO from the shells of chicken eggs, oysters, lobsters, and mussels, reporting a yield greater than 90%. A biodiesel yield of 97.9% was reported by Gaide et al. [11], who extracted CaO from eggshells. Jitjamnong et al. [12] also extracted CaO from eggshells and reported a biodiesel yield of 94%.

Following the revolutionary work of Haruta [13] and Hutchings [14], immense research has been conducted on the role of gold nanoparticles in catalysis. The amazing catalytic activity of gold has verified its use in a variety of chemical reactions such as the hydrogenation of organic substrates [15,16], selective oxidation [17,18], acetylene hydrochlorination [19], and water gas shift reaction [20]. Despite gold’s use as an alloy with other metals such as silver, copper, palladium, and platinum, gold nanoparticles display extraordinary catalytic activities on their own [21]. Several researchers have established that the catalytic activity of gold is influenced by a number of factors: (i) the method used for synthesis [22], (ii) the shape and size of gold nanoparticles [23], (iii) the interface interactions between the gold nanoparticles and the support used [24], (iv) the nature of the nanoparticles’ support [25,26], and, lastly, (v) the oxidation states of gold in the used catalyst [27]. Achieving high dispersion of a noble metal catalyst on support has been an important issue in the area of heterogeneous catalysis [28,29]. Mesoporous silica materials have been one of the most used/studied supports for gold nanoparticles in the literature. These materials are reported to have a controllable porous structure [30], high specific surface area and pore volume [31], exceptional mechanical properties [32], and high thermal stability. These properties make them excellent catalyst supports since they provide a good dispersion of synthesised nanoparticles and promote substrates’ ability to access active sites [33]. Supporting gold nanoparticles on a solid support such as mesoporous silica make it possible for the solid catalyst to be recycled, which could be cost-effective [34].

Gold nanoparticles supported on mesoporous silica have been a useful catalyst in a wide range of applications but mostly in oxidation reactions [34,35,36,37,38]. Wu et al. [34] used gold nanoparticles supported on mesoporous silica in the selective oxidation of cyclohexane. These researchers discovered that the added gold nanoparticles played a critical role in promoting the activation of oxygen (O_2_) molecules and in the formation of surface-active O_2_ species. Trayford et al. [39] used this catalyst in a medical-based application focused on stem cell therapy. They discovered that gold nanoparticles on a mesoporous silica support permit multimodal imaging and simultaneous reactive oxygen species sensing of stem cells, providing a promising tool for in vivo stem cell tracing. Chen et al. [40] used gold nanoparticles intercalated on mesoporous silica as a redox catalyst to reduce methylene blue. They reported high catalytic rates even after recycling the catalyst 10 times. Mesoporous silica derived from agricultural residues has also been used as a support for gold nanoparticles. Li et al. [41] synthesised gold nanoparticles on a rice husk-derived mesoporous silica support and used it as a catalyst to reduce 4-nitrophenol. They reported enhanced catalytic activity due to the presence of gold nanoparticles.

This study reports the application of gold nanoparticles supported on a sugarcane leaves-derived silica xerogel as a catalyst in the production of biodiesel. Sugarcane leaves are usually burned pre-sugarcane harvest, which results in environmental pollution and health issues for sugarcane farm workers and exposes the community at large [42,43]. Several studies report the usage of waste or agricultural residues as catalysts in the production of biodiesel. This study, however, uses a catalyst derived from an agricultural residue with gold nanoparticles added to it to enhance biodiesel productivity. Gold nanoparticles have a large surface area-to-volume ratio. Their incorporation into a transesterification reaction was expected to provide more reaction sites, leading to more chemical reactivity. Canola oil was used as a triglyceride and methanol as an alcohol of choice. To the best of our knowledge, no application of sugarcane leaves-derived silica-gold nanoparticles is reported in the literature.

## 2. Results and Discussion

### 2.1. Catalyst Characterisation

#### 2.1.1. XRF Analysis

Chemical analysis of the synthesised silica-gold nanoparticles xerogel catalyst was performed using XRF analysis. Table 1 shows that silica was the main constituent with a percentage of 91.64 wt%, followed by sodium oxide with a percentage of 6.37 wt%, then gold with 1.97 wt%. Both CaO and K_2_O only had percentages of 0.01 wt% each. The 0.01 wt% of CaO and K_2_O indicates the sample’s high purity since these are considered impurities. The added amount of gold (2%) was successfully preserved since 1.97 wt% of gold was present in the silica-gold xerogel catalyst sample, indicating the effectiveness of the synthesis method. Since this catalyst is a solid-basic catalyst, a healthy amount of sodium is present in the xerogel to facilitate the catalyst application in the intended transesterification reaction.

#### 2.1.2. XRD Analysis

**Figure 2:**
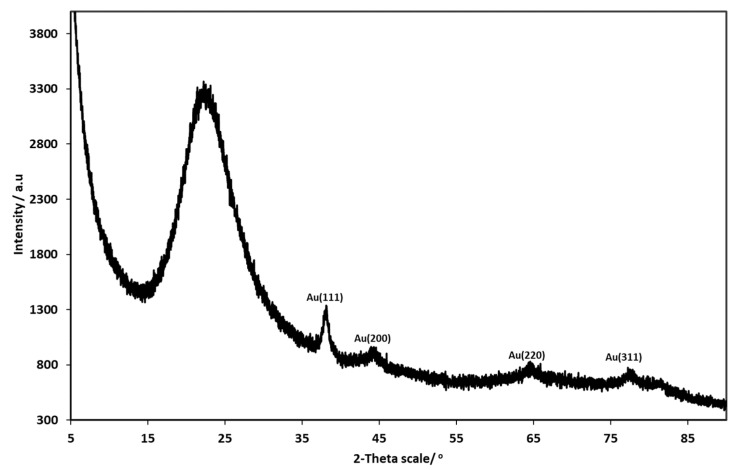
XRD pattern of silica-gold nanoparticles xerogel.

Different phases present in the silica-gold nanoparticles xerogel were identified through XRD analysis, as displayed in Figure 2. The broad reflex with a Bragg angle of 2θ between 15° and 30° confirms the presence of amorphous silica, indicating the existence of silica particles in the sample [44,45]. The reflexes at 38.21, 44.45, 65.04, and 77.65° correspond to (1 1 1), (2 0 0), (2 2 0), and (3 1 1) Miller indices, respectively [46]. These lattice planes represent the standard face-centred cubic (FCC) phase of metallic gold structures. These are identical to the ones reported on the joint Committee on Powder Diffraction Standards (JCPDS no 04-0784, USA) for the standard gold metal (Au^o^), which suggests the formation of crystalline gold nanoparticles [46,47,48].

#### 2.1.3. Textural Properties

**Figure 3:**
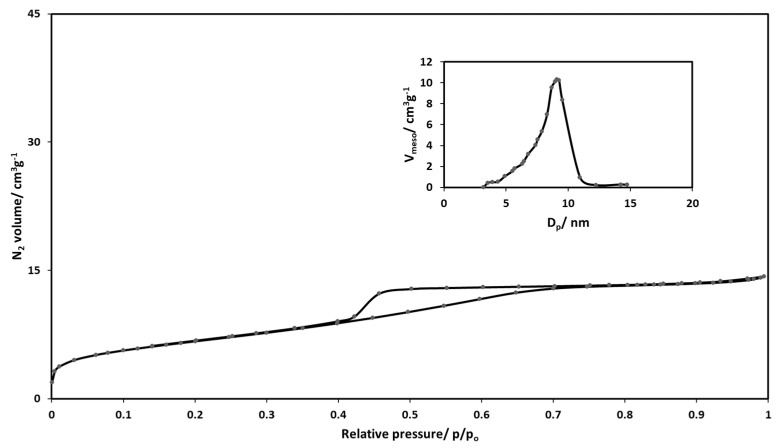
Nitrogen sorption isotherm of silica-gold xerogel catalyst from sugarcane leaves with its corresponding pore diameter distribution.

The nitrogen physisorption isotherm for the silica-gold nanoparticles xerogel is displayed in Figure 3. According to the IUPAC classification, this type of silica matrix belongs to a type IV isotherm, which is typical of mesoporous materials. Hysteresis can be observed in the isotherm pattern where capillary condensation follows the initial monolayer–multilayer adsorption on the mesopore walls [7,49]. The hysteresis loop has a closure point at 0.42, which can be further classified as H2 [50]. This type of isotherm is usually observed in gels of inorganic oxides where both pore size distribution and shape are not well defined [49].

Table 2 shows the textural properties of silica-gold nanoparticles xerogel catalysts such as apparent surface area, pore diameter, and pore volume together with the properties of silica xerogel without gold nanoparticles. The apparent surface area of the silica-gold xerogel catalyst is 54% less than that of silica xerogel without gold nanoparticles due to the immobilization of gold nanoparticles on the pore surface, which causes a distortion effect [51]. The pore volume decreased from 1.26 cm^3^g^−1^ to 0.89 cm^3^g^−^1. The pore diameter distribution according to the BJH method (Barret-Joyner-Halenda) [52] is presented in Figure 3. The pore diameter of the silica-gold xerogel catalyst is 8.9 nm while that of silica-xerogel is 7.5 nm. The unexpected increase in pore diameter might be due to smaller pores being blocked by gold nanoparticles. The peak position of the distribution curve was used for pore sizes. The majority of pores are in the lower mesopore region, as shown in Figure 3.

#### 2.1.4. Transmission Electron Microscopy

**Figure 4:**
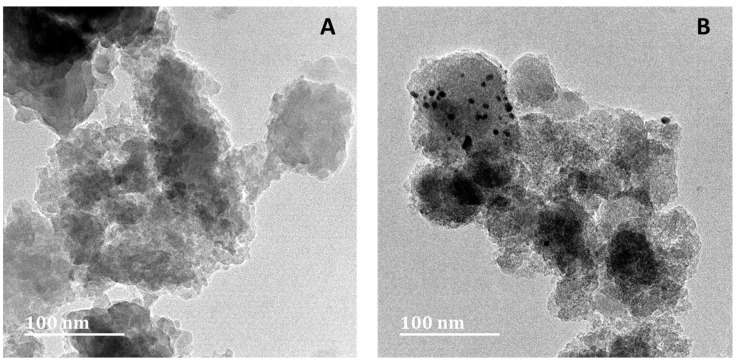
TEM images of silica xerogel (**A**) and gold nanoparticles deposited on a silica support (**B**).

TEM was employed to analyse the size, distribution, and morphology of the synthesised nanoparticles. The images of silica xerogel and silica xerogel with deposited gold nanoparticles are presented in Figure 4A and Figure 4B, respectively. It can be observed that both images show silica xerogel particles with no defined shape accompanied by some degree of agglomeration, which forms larger particles. This phenomenon is reflected in the mesopores identified in the nitrogen adsorption–desorption isotherm. Gold nanoparticles present in Figure 4B are unevenly distributed and exhibit a predominantly spherical morphology. This behaviour is due to a majority of the particles aggregating to create larger particles, resulting in considerable agglomeration.

### 2.2. Exploring the Optimum Reaction Conditions

#### 2.2.1. Impact of Reaction Time on Biodiesel Production

**Figure 5:**
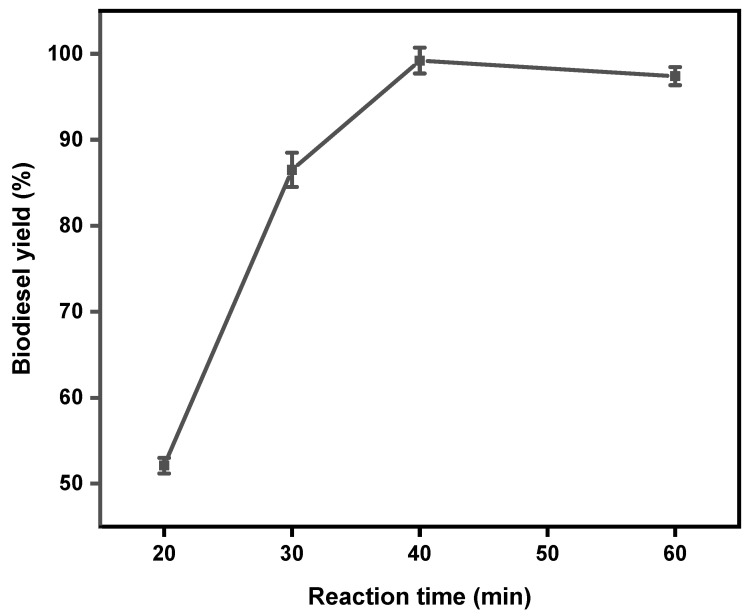
The effect of reaction time on the yield of biodiesel. Reaction conditions: 3 wt% catalyst loading, 65 °C, and 6:1 methanol-to-oil ratio.

The effect of reaction time was studied under reaction conditions of 3 wt% catalyst loading, 65 °C reaction temperature, and 6:1 methanol-to-oil ratio. Different reaction times: 20, 30, 40, and 60 min were used for the study. At 20 min, the biodiesel yield was the lowest at 52.1% and the yield increased as the time increased, reaching a maximum yield of 99.2% after 40 min. Further increasing the time to 60 min slightly decreased the yield to 97.4%. This might be caused by the reverse transesterification reaction after the reaction had reached equilibrium since the transesterification reaction is reversible, as shown in Figure 5 [7]. Bazhdan et al. [1] used olive oil in their transesterification and reported a yield reduction above the equilibrium point.

The best yield was obtained at 3 wt% catalyst loading, 65 °C reaction temperature, 6:1 methanol-to-oil ratio, and 40 min reaction time. As seen in Table 3, the addition of gold nanoparticles caused the optimum reaction time to reduce from 60 min to 40 min. The yield also increased from 96.9 to 99.2%.

#### 2.2.2. Impact of Catalyst Loading on Biodiesel Production

**Figure 6:**
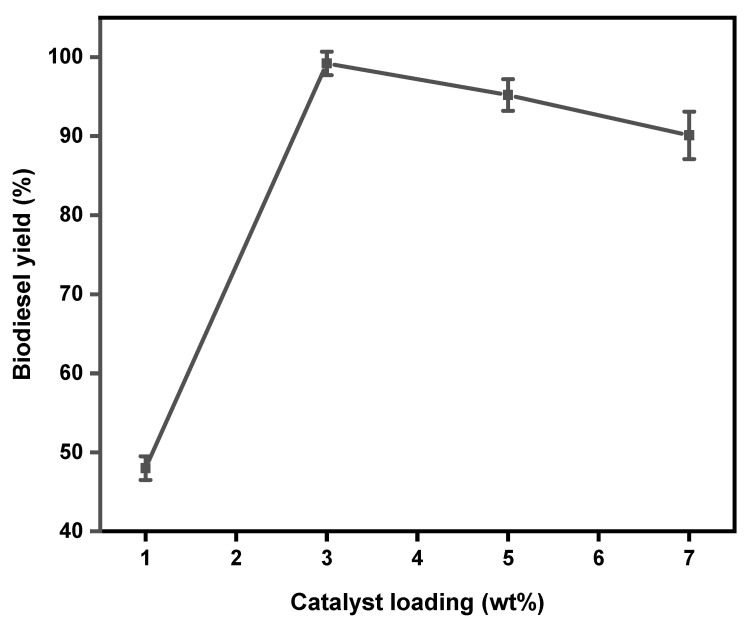
The effect of catalyst loading on the yield of biodiesel. Reaction conditions: 65 °C, 6:1 methanol-to-oil ratio, and 40 min reaction time.

The amount of catalyst used strongly affected the transesterification reaction since no reaction occurred in the absence of a catalyst. Generally, the more effective the catalyst is, the smaller the required dosage of the catalyst [1]. Figure 6 represents the results obtained from evaluating the effect of catalyst loading on biodiesel production. Different catalyst loadings: 1, 3, 5, and 7 wt% were investigated at 65 °C, 40 min, and a 6:1 methanol-to-oil molar ratio. As seen in Figure 6, the biodiesel yield started low at 47.4% when 1 wt% catalyst loading was employed. However, it rapidly increased to 99.2% when 3 wt% catalyst was used. It was noticed that when the catalyst loading was increased to 5 wt% the yield slightly decreased to 96.1%. A further increase in the catalyst dosage to 7 wt% caused a significant decrease in biodiesel yield and the reaction mixture became noticeably viscous. According to several researchers [1,7,53], the decrease in biodiesel yield as catalyst dosage increases is caused by the increased viscosity of the reaction mixture, resulting in restricted diffusion between the oil and methanol. The optimum biodiesel yield was obtained when 3 wt% of catalyst loading, 65 °C reaction temperature, 40 min reaction time, and a 6:1 methanol-to-oil ratio were employed.

#### 2.2.3. Impact of Methanol-to-Oil Ratio on the Production of Biodiesel

**Figure 7:**
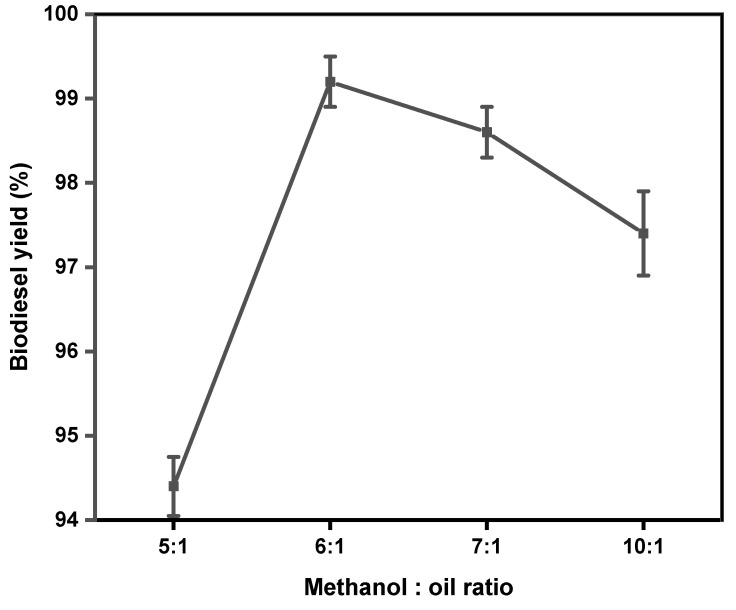
The effect of methanol-to-oil ratio on biodiesel yield. Reaction conditions: 65 °C, 40 min and 3 wt% catalyst loading.

Methanol-to-oil ratio plays an important role in biodiesel production since it determines the conversion of triglycerides to methyl esters, which affects the yield and production cost of biodiesel [1,7]. According to the transesterification reaction in Figure 1, the stoichiometric methanol-to-oil ratio is 3:1 to produce 3 mol of biodiesel and 1 mol of glycerol [54]. However, since transesterification is a reversible reaction, an excess amount of methanol is used to improve the equilibrium conversion, thereby driving the reaction to the right side to yield more biodiesel [53]. The effect of the methanol-to-oil ratio was tested at a 65 °C reaction temperature, 3 wt% catalyst loading, and 40 min reaction time. Different methanol-to-oil ratios of 5:1. 6:1, 7:1, and 10:1 were tested. The results are displayed in Figure 7.

It can be observed that increasing the methanol-to-oil ratio from 5:1 to 6:1 increased the biodiesel yield. This finding was reported by Boro et al. [55], who considered it to be due to the formation of methoxy species on the surface of a catalyst, which causes the reaction to shift towards the forward direction. However, increasing the methanol-to-oil ratio to 7:1 resulted in a yield reduction. A further increase to 10:1 significantly decreased the biodiesel yield. This phenomenon may be related to the high molar ratio of methanol-to-oil interfering with glycerol separation due to an increase in glycerol’s solubility in excess methanol. Glycerol helps shift the equilibrium back to the left when present in a solution, reducing the yield of esters and encouraging the balance to shift in the opposite direction, thereby forming mono, di, and triglycerides, which decrease the production of esters [56]. The effect of increasing the methanol-to-oil ratio has been reported by other researchers in the literature [7,8,56,57,58] and all of these authors reported a decrease in biodiesel yield when the methanol-to-oil ratio is increased after the biodiesel optimum yield is reached.

#### 2.2.4. Catalyst Reusability Study

**Figure 8:**
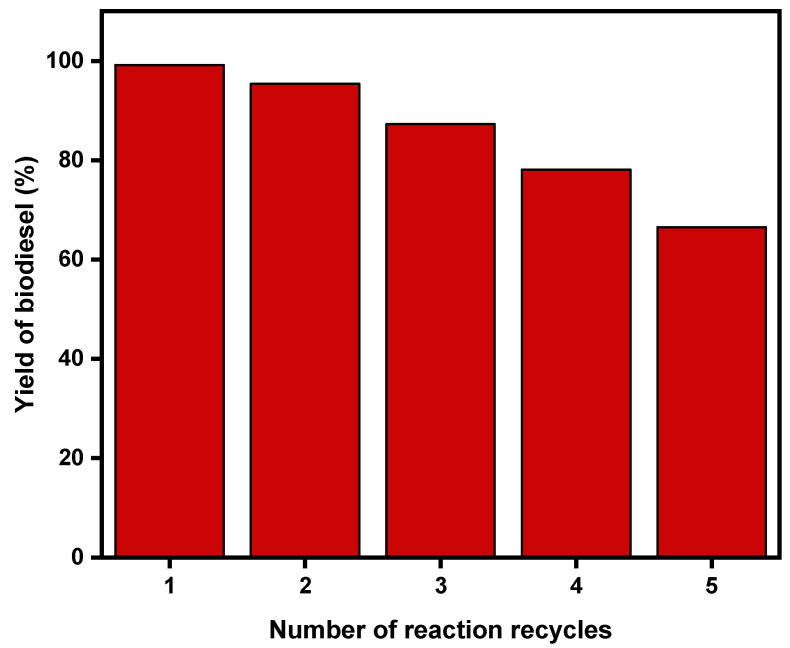
Reusability of silica-gold nanoparticles in the production of biodiesel.

Solid catalysts have an added advantage over homogenous catalysts because they can be reused several times, which reduces biodiesel production costs. Optimum conditions of 40 min reaction time, 65 °C reaction temperature, 3 wt% catalyst loading, and 6:1 methanol to oil ratio were used to study the catalyst’s recyclability potential. After each use, the catalyst was washed with methanol and then dried at 80 °C in the oven overnight. After the catalyst was used for the second time, the yield dropped to 95.4% and decreased with each use until it reached 66.5% after the catalyst was used for the fifth time. Figure 8 demonstrates the recyclability potential of silica-gold xerogel as a biodiesel catalyst.

### 2.3. Biodiesel Analysis

**Figure 9:**
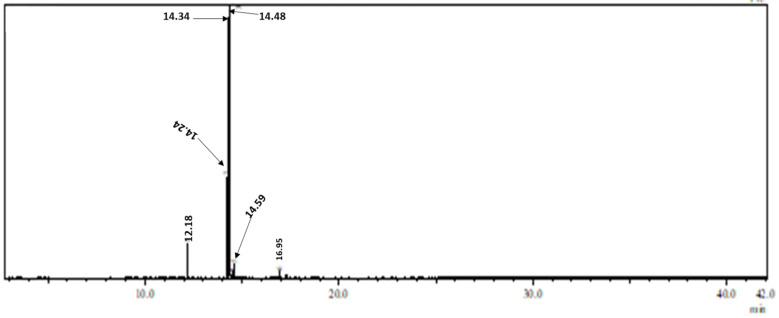
GC-MS chromatogram of biodiesel produced from methanol and canola oil using silica-gold nanoparticles as a catalyst.

The chemical composition of the produced biodiesel was determined using GC-MS. The GC-MS chromatogram is represented in Figure 9 and the six peaks within it are represented in Table 4. The peaks were identified through comparison with reported data and profiles found in NIST and Wiley GC-MS libraries.

Monounsaturated fatty acid methyl ester, which consists of methyl oleate and methyl gondoate, was found to be the most dominant FAME, with a total composition of 72.23 wt%. By contrast, the total composition of saturated FAME was the least present with a composition of 7.52 wt%.

The quality of biodiesel was evaluated by measuring its physical properties, such as density, viscosity, flash point, cloud point, and pour point, as indicated in Table 5.

All the physical properties measured for the produced biodiesel fall within the standard of American and European biodiesel. The produced biodiesel can be used at temperatures as low as −5 °C without the need for blending with fossil diesel. The flashpoint was found to be 167 °C, which is relatively high. This makes the produced biodiesel attractive in terms of storage, handling, and transportation. Since the measured physical properties of the produced biodiesel fall within the internationally accepted standards, the biodiesel quality can be endorsed as satisfactory.

## 3. Conclusions

This study focused on the transesterification of canola oil where methanol was used as a solvent in the presence of an agricultural-based catalyst. A sol-gel was used to deposit gold nanoparticles on a silica support from sugarcane leaves to facilitate the reaction. The resulting silica-gold xerogel had gold nanoparticles that were spherical in nature but agglomerated to form bigger particles. The catalyst was in a solid form and remained solid throughout the transesterification reaction as different parameters such as reaction time, reaction temperature, methanol to oil ratio, and catalyst loading were studied. The outcome indicated that the optimum biodiesel yield was 99.2% when reaction conditions were 3 wt% catalyst loading, 40 min reaction time, 65 °C reaction temperature, and 6:1 methanol to oil molar ratio. The biodiesel yield dropped to 66.5% after five consecutive cycles. The produced biodiesel had both saturated and unsaturated fatty acid methyl esters with monounsaturated FAME being the most dominant at 72.23 wt%. The biodiesel was also discovered to fall within European and American standards and was suitable for use at cold temperatures without the need for blending. The addition of gold nanoparticles to silica slightly improved the transesterification reaction since a higher yield was obtained and the optimum reaction time was shorter than when using silica xerogel without gold nanoparticles. The reported catalyst might be more suitable for reactions with a significantly lower biodiesel yield, justifying the addition of gold nanoparticles.

For future work, the method can be improved in such a way that the produced biodiesel does not need a washing step to save water.

## 4. Materials and Methods

### 4.1. Materials

Citric acid, polyvinyl alcohol (average molecular weight, 9000–10,000), gold (III) chloride trihydrate (99.9%), sodium borohydride (99.9%), and sodium hydroxide were all purchased from Merck (Lethabong, South Africa). Methanol (analytical grade), phenolphthalein indicator, and absolute ethanol (99.9 AR) were purchased from Reflecta Laboratory Supplies (Johannesburg, South Africa). Canola oil with a free fatty acid (FFA) content of 0.089% was acquired from Woolworths (Durban, South Africa) and sugarcane leaves were obtained from sugarcane farmers in Verulam (South Africa). All chemicals were used as obtained.

### 4.2. Methods

#### 4.2.1. Synthesis of Sodium Silicate

Silica xerogel was synthesised from amorphous biogenic silica, which was produced from sugarcane leaves through a thermochemical method reported by Maseko et al. [46]. Biogenic silica was mixed with 1 M sodium hydroxide and the mixture was refluxed for 2 h at 80 °C. The resulting sodium silicate solution was gravity-filtered and cooled to room temperature. Once the sodium silicate was cooled to room temperature, it was used to synthesise silica-gold nanoparticles.

#### 4.2.2. Synthesis of Silica-Gold Nanoparticles Xerogel

3M citric acid was then added to the solution until a pH of 9.5 was reached. Gold (III) chloride trihydrate together with polyvinyl alcohol were added to the solution and the mixture was stirred for 30 min. After 30 min, sodium borohydride (NaBH_4_) was added and the solution was vigorously stirred for another 30 min. The earlier solution of 3M citric acid was added again to the mixture until a pH of 8.7 was reached when a gel started forming. The resulting gel was covered and allowed to age for 8 h at room temperature before being broken and washed with deionized water three times. The silica-gold nanoparticles gel was subjected to solvent exchange, as reported by Maseko et al. [59], where ethanol-water solutions were introduced to the washed gel in varying ratios of 1:3, 1:1, and 1:0. These mixtures were allowed to sit overnight to facilitate solvent exchange, during which water molecules were gradually substituted for ethanol molecules. Following the solvent exchange process, the gel was dried in an oven at 80 °C for a duration of 24 h. Once the drying was complete, the sample was retrieved from the oven and stored in a desiccator for future use.

#### 4.2.3. Determination of Free Fatty Acid Content of Canola Oil

A volume of 50 mL ethanol was added to a conical flask prior to adding a few drops of the phenolphthalein indicator. The ethanol was neutralised by titrating it with 0.1 N sodium hydroxide until a change of colour was observed. The neutralised ethanol was then mixed with 10 g of canola oil. The resulting solution was heated until the oil was completely dissolved in ethanol. Once the oil had completely dissolved in ethanol, the resulting solution was titrated with 0.1 N sodium hydroxide while vigorously agitated until an end-point was reached. The sodium hydroxide volume used to titrate the ethanol-canola oil solution was then used to determine the acid value and free fatty acid content. 

#### 4.2.4. Production of Biodiesel

The transesterification reaction was conducted in a 500 mL two-neck round bottom flask equipped with a thermometer in a reflux setup. An amount of 50 g canola oil was added to the round bottom flask and heated to 65 °C. After the temperature reached 65 °C, 1, 3, or 5 wt% of the silica-gold nanoparticles catalyst was added followed by methanol in a 5:1, 6:1, 7:1, or 10:1 methanol:oil ratio. The temperature was maintained at 65 °C until optimum reaction conditions were reached. Once the reaction had taken place for a specific time: 20, 30, 40, or 60 min, the heating mantle was turned off. The resulting yellowish liquid was poured into a large beaker and kept in a fume hood to facilitate biodiesel separation. The silica-gold nanoparticles catalyst remained in the round bottom flask since it was in solid form. After methanol evaporated while the solution was in the fume hood, two layers were separated: the top biodiesel layer and the bottom glycerol layer. After layer separation, the resulting product was poured into a separation funnel. The bottom glycerol layer was discarded while the biodiesel layer was washed with hot water and later placed in an oven to dry for 24 h.

### 4.3. Characterisation

Textural properties were measured using ASAP 2010, Micromeritics, Norcross, 157, GA, USA. An X-ray fluorescence analysis S4 Explorer, WDXRF Bruker, (Karlsruhe, Germany) was used for the chemical analysis of the silica-gold nanoparticles xerogel catalyst. The phase identification was measured using a Seifert XRD 7 apparatus (Ahrensburg, Germany) equipped with Ni-149 filtered, Cu-Kα radiation (λ = 1.54 Ȧ) [59]. The transition electron microscopy (TEM) images were obtained from JEOL 2100 HRTEM (Tokyo, Japan). The fatty acid methyl ester (FAME) concentrations, expressed as the purity of the biodiesel product, were measured using a Shimadzu GC-MS-QP2010 SE (Kyoto, Japan). Helium served as the carrier gas, maintained at a flow rate of 1.0 mL/min. The injector and detector temperatures were established at 250 °C. The oven temperature commenced at 120 °C, subsequently increased to 230 °C, and ultimately elevated to 300 °C at a rate of 8 °C/min. An aliquot of 0.2 mL of dichloromethane (DCM) was injected at a split ratio of 1:50. The mass spectrometer was configured to scan within the range of 40–700 *m*/*z*. An Anton Paar viscometer (Graz, Austria) was used to measure the viscosity and density of biodiesel.

## Figures and Tables

**Table 1:** Chemical analysis of silica-gold nanoparticles xerogel catalyst.

Constituent	Silica-Gold Nanoparticles (wt%)
SiO_2_	91.64
Na_2_O	6.37
CaO	0.01
K_2_O	0.01
Au	1.97

**Table 2:** Textural properties for silica and silica-gold xerogel catalyst determined via nitrogen physisorption.

Catalyst	SBET (m^2^g^−1^)	Total Pore Volume (cm^3^g^−1^)	Pore Diameter (nm)
Silica xerogel	668	1.26	7.5
Silica-gold xerogel	309	0.89	8.9

**Table 3:** Comparison of xerogel with and without gold nanoparticles as a catalyst in biodiesel production.

Catalyst Type	Yield (%)	Reaction Time (min)
Silica Xerogel	96.9	60
Silica xerogel-Au nanoparticles	99.2	40

**Table 4:** Composition of fatty acid methyl esters produced from transesterification of canola oil with methanol using silica xerogel deposited with gold nanoparticles.

Retention Time (RT) (min)	Fatty Acid Methyl Ester	Fatty Acid Methyl Ester Type	Composition (wt%)
12.18	Methyl palmitate	Saturated	5.32
14.24	Methyl linoleate	Poly unsaturated	19.50
14.34	Methyl oleate	Mono unsaturated	70.96
14.48	Methyl linolenate	Poly unsaturated	0.75
14.59	Methyl stearate	saturated	2.20
16.95	Methyl gondoate	Mono unsaturated	1.27

**Table 5:** Physical properties of biodiesel produced from sugarcane leaves-derived silica xerogel catalyst.

Physical Property	Biodiesel	ASTM D 6751	EN 14214
Density (kg/m^3^)	877 ± 1.5	870–900	860–900
Kinematic viscosity (mm^2^/s)	4.4 ± 0.06	1.9–6.0	3.5–5.0
Cloud point (°C)	−1 ± 1.0	−3–15	^a^
Pour point (°C)	−5 ± 1.0	−15–10	^a^
Flash point (°C)	167 ± 0.6	>130	>120

^a^ data not available.

## Data Availability

The original contributions presented in this study are included in the article. Further inquiries can be directed to the corresponding author.
